# Burying poultry carcasses on farms as a disposal option in crisis situations: learnings and perspectives from a field study during an avian influenza epizootic in France

**DOI:** 10.1016/j.psj.2025.104806

**Published:** 2025-01-13

**Authors:** R. Souillard, M. Salines, C. Martenot, C. Le Maréchal, L. Bonifait, A. Scoizec, R. Thomas, I. Pierre, S. Rouxel, G. Venet, C. Mourrieras, B. Grasland, S. Le Bouquin

**Affiliations:** a Ploufragan-Plouzané-Niort Laboratory, Epidemiology Health and Welfare Unit, French Agency for Food, Environmental and Occupational Health and Safety (ANSES), BP53 22440 Ploufragan, France; b Ploufragan-Plouzané-Niort Laboratory, Avian & Rabbit Virology, Immunology & Parasitology Unit, French Agency for Food, Environmental and Occupational Health and Safety (ANSES), BP53 22440 Ploufragan, France; c Ploufragan-Plouzané-Niort Laboratory, Hygiene and Quality of Poultry and Pig Products Unit, French Agency for Food, Environmental and Occupational Health and Safety (ANSES), BP53 22440 Ploufragan, France; d Veterinary Services of the Vendée Department, BP 90795 85020 La Roche-sur-Yon, France

**Keywords:** Carcass burial, Avian influenza, *Clostridium botulinum*, *Salmonella*

## Abstract

Appropriate disposal of dead farming animals is required to guarantee effective disease control while protecting the environment. In crisis situations, alternatives to rendering can be used, including on-farm burial. The objectives of this study were to: (i) describe the burial and monitoring protocols used on poultry farms in France in response to major avian influenza outbreaks; (ii) assess the effectiveness of the burial protocol, in terms of both technical and biosecurity aspects, and microbiological, physical and chemical changes of the buried materials and the environment over time; (iii) provide recommendations for future burial and follow-up protocols. Five on-farm burial sites were monitored between March 2022 and March 2023, with at least four visits per farm. In addition to visual observations, soil, leachate, air and drilling water samples were collected, as well as boot swabs on/near the pit or on carcasses. For all five farms, microbiological analyses were performed to detect avian influenza virus (AIV), *Clostridium botulinum* and *Salmonella spp.* At one site, sampled drilling water was analysed to describe its physical and chemical properties. Visual anomalies were found at the sites over time, such as subsidence of the pits, presence of traces of wild and domestic animals, and rising to the surface of pieces of carcasses and feathers. AIV RNA was detected at all burial sites and in 4 % (8/201) of the collected samples. Viral genome was found up to nine months after burial on one farm. *Clostridium botulinum* was detected in 16 % (19/117) of the samples, whereas all samples tested negative for *Salmonella* spp. (0/109) at all sites and at all sampling points. All drilling water samples were compliant with drinking water standards. Our assessments demonstrated how the burial pits changed over time and the need to monitor them regularly so that corrective measures can be taken, if needed. In conclusion, our study can be used as a baseline for preparing better burial and follow-up protocols for future crisis situations. We recommend to standardise trench size and depth, to add mounding soil to the top of the pit and to set up a fence around. Proper pre-planning in peacetime will make it easier to meet the challenges associated with the management of repeated, high-frequency crises or crises of a new nature.

## Introduction

Animal mortality in livestock farming is unavoidable, regardless of the species. On-farm mortality can be linked to a number of causes, among them infectious diseases or non-infectious reasons such as predation, accidents or natural disasters. Under European regulations (Regulation (EC) No 1069/2009), dead farming animals are considered to be animal by-products and categorised as category 1 or category 2 by-products depending on the species concerned and/or the substances they may contain (Anonymous, 2009). Appropriate disposal of dead animals is necessary, in particular to guarantee effective disease control while protecting the environment. The European regulation describes several disposal methods for each animal by-product category. Rendering is defined as the collection, handling, post-collection storage, treatment or disposal of one or more animal carcasses or parts thereof or other animal materials (Article L 226-2 of the French Rural and Sea Fishing Code) (Anonymous, 2024). Although rendering is most often the preferred disposal method, it may have some limitations in specific situations. For example, exceptional circumstances may lead to an accumulation of carcasses on a farm, and/or a rendering plant exceeding its capacity: natural disasters such as floods or heat waves; and detection of a pathogen on farms inducing massive mortality and/or culling measures (e.g. avian influenza (AI), foot and mouth disease (FMD) or African swine fever (ASF)). In these situations, several alternatives to rendering can be considered, such as on-farm pyre, on-farm burial, mass burial, composting, anaerobic digestion or alkaline hydrolysis (Chowdhury et al., 2019). On-farm carcass burial has been used as a method of carcass disposal in several countries and in several major disease eradication efforts, including the 2001 FMD outbreak in the United Kingdom, the 2002 outbreak of exotic Newcastle disease in Southern California, USA, as well as the 1984 and 2002 AI outbreaks in Virginia, USA (Consortium, 2004). Even though it is cost-effective, carcass burial can have detrimental environmental effects, e.g. water quality issues, and may pose the risk of disease agents persisting in the environment. For example, monitoring of groundwater quality at mass burial sites for pigs infected with FMD virus in Taiwan in 1997 showed contamination by chemical indicators measured (nitrites, nitrates, sulphates, etc.) (Hseu and Chen, 2017). Similar results were obtained from monitoring carried out in South Korea following mass burials of poultry and pigs in connection with AI and FMD epizootics (Kaown et al., 2015; Koh et al., 2019; Kwon et al., 2017). Ritter and Chirnside (1995) also observed high ammonia and nitrate concentrations in ground water collected from wells adjacent to poultry burial pits. Glanville (2000) also reported similar observations from monitoring wells installed near turkey and swine carcass burial pits. Regarding the risk of pathogens remaining in the environment after burial, water contamination by bacteria (coliforms in particular) has also been described, but the results of these studies are not entirely consistent. Some authors have indicated that microbiological contamination had occurred (Kim, and Kim, 2012; Koh et al., 2019), and was long-lasting (Hseu and Chen, 2017), while others have indicated that this contamination was negligible (Joung et al., 2013; Ritter and Chirnside, 1995).

Microbiological contamination seems to decrease rapidly with distance from the burial site. For example, Myers et al. (1999) reported low concentrations of coliforms and *Salmonella* spp. in observation wells surrounding burial pits, concluding that bacteria did not move more than 30 meters laterally in groundwater. Similarly, in a survey of poultry disposal pits, Ritter and Chirnside (1995) found the average concentrations of faecal coliforms and faecal streptococci in water samples to be relatively low (24 CFU.100 mL^-1^ and 3 CFU.100 mL^-1^, respectively), with many samples testing negative. Virus survival was also studied, especially in pigs in the context of ASF, in different matrices. The European Food Safety Authority (EFSA) reported that the ASF virus remains viable in the carcasses of wild boars contaminated experimentally with infected blood for 81 days (summer-autumn) after burial in soil to a depth of 12 cm, and for 192 days when they were left on the soil surface (EFSA, 2014). A recent study by Zani et al. (2020) in which carcasses of wild boars infected with ASF were dug up and analysed (bone marrow, various tissues and soil samples) also showed that viral genomes could be found in 17 of the 20 carcasses analysed, but no viral isolation was possible. In addition, viral genome was also found in seven soil samples.

**Fig. 1:**
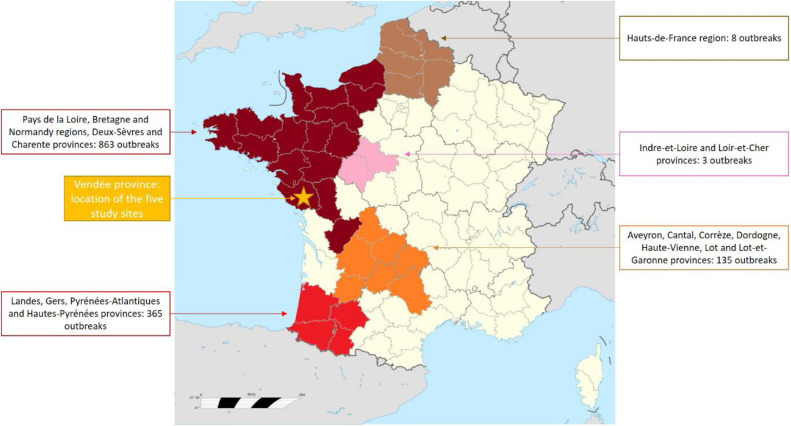
Provinces and regions affected by HPAI in 2021-2022 and location of the five study sites Separate JPEG file.

In the European Union, on-farm burial is authorised by the European regulation as an exception to the general provisions, e.g. to prevent the availability or capacity of a rendering plant within a region or a Member State from being a limiting factor in the control of a disease (Anonymous, 2009). This derogation was used in France during the 2021–2022 highly pathogenic avian influenza (HPAI) crisis. HPAI is a major contagious pathogenic viral disease that poses a significant threat to animal health, human health, the global economy and farming sustainability. In recent years, the world has experienced unprecedented HPAI H5N1 epidemics since the emergence of clade 2.3.4.4b panzootic viruses, affecting more than 70 countries across the world. These outbreaks resulted in the death of over half a billion poultry worldwide, either due to the virus itself or to attempts to stop the virus spread through culling, and in massive deaths in wild bird populations (FAO, 2024; WOAH, 2024). Like many other countries, France has been hard hit by these epizootics. During the 2021–2022 season, France officially declared 1,378 HPAI outbreaks on poultry farms, 72 cases in wildlife and 35 cases in backyard flocks (Le Bouquin et al., 2023). In contrast to previous outbreaks, this epizootic had the particularity of successively affecting three major poultry production areas in France. After a first “wave” in the duck production area in south-western France with 356 outbreaks, other production regions experienced significant increases in the number detections at the end of February 2022 in western France (Pays de la Loire region). This second wave was responsible for 863 outbreaks. The virus spread on an unprecedented scale, both in terms of number of outbreaks and speed of disease spread. The Vendée province (Fig. 1) was the most seriously affected (522 outbreaks on poultry farms as of 23 May 2022). For several weeks, the explosion in the number of outbreaks generated very high daily tonnages of carcasses due to mortality linked to the virus itself, but also to culling of affected poultry and preventive culling of the surrounding poultry farms. This led to the saturation of rendering plant capacity. For example, of the 20,000 tonnes of carcasses produced in Vendée, rendering plants were able to process just under a third. In this context, the French Competent Authority decided to authorise, as describe in the national health emergency plan, temporary or definitive burial of poultry carcasses on HPAI affected farms to guarantee disease spread control and carcass disposal. In this crisis setting, a study was carried out (i) to describe the burial and monitoring protocols used at five on-farm definitive burial sites; (ii) using a one-year longitudinal follow-up, to assess the effectiveness of the burial protocol, in terms of both technical and biosecurity aspects, and microbiological, physical and chemical changes of the buried materials and the environment over time; and (iii) to provide recommendations for future burial and follow-up protocols to be implemented.

## Materials and methods

### Studied burial sites and burial protocol

Five on-farm burial sites were monitored over the period from March 2022 to March 2023. All five farms were HPAI affected and were located in the Vendée province (Fig. 1). Monitoring began for all farms in the second half of March. The choice of the burial site location on the farm was decided by an approved hydrogeologist and local veterinary authorities. Their mission was to assess the suitability of the land for a burial pit based on the following criteria:

• easily accessible site, where the ground is level or has a slope lower than 5 %
• ground that is easy to dig to a depth of at least 2 metres (m)
• no wet areas or depressions
• bottom of the pit above the water table
• site located:
  ○ more than 50 m from livestock buildings
  ○ more than 100 m from any dwelling
  ○ more than 100 m from springs, watercourses, wells/bores and bodies of water
  ○ outside the close protection perimeter of a public drinking water catchment
• site located away from field drainage networks (ditches, drains) and buried pipes (water/gas/electricity/fibre optics, etc.)
• site not subject to construction plans, as no excavation could be carried out at the site for at least 5 years
• burial area protected for several months from any access by people (6 months) and animals (9 months): no crops, poultry, etc. planted/established during this period.

If all the criteria were met, the approved hydrogeologist carried out soundings before authorising digging of the burial pit.

**Fig. 2:**
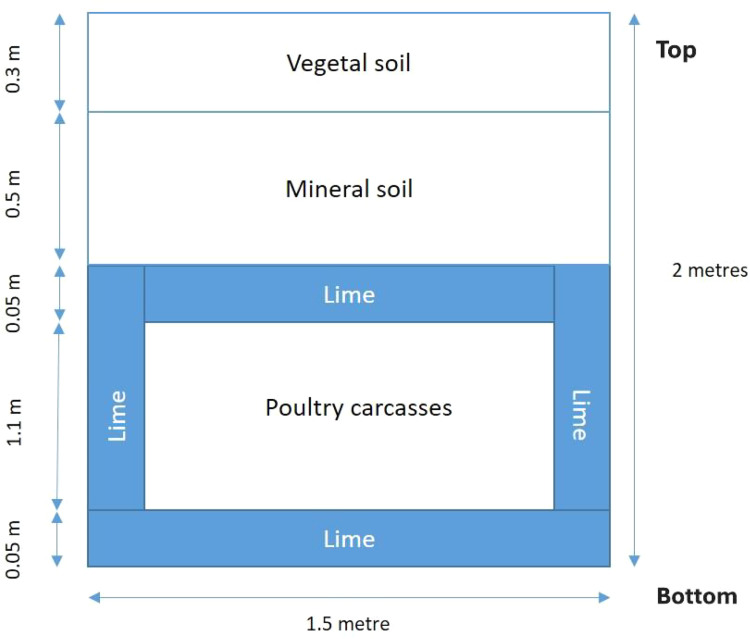
Cross-sectional view of the pit Separate JPEG file.

Once authorisation had been given, the excavation work was carried out by a public works company. The filling of the pit with lime and carcasses was done by the farmer. The entire worksite was supervised by the veterinary services. All selected sites were definitive burial sites. The dimensions of the pits were 20 m long by 1.5 m wide by 2 m deep. For each pit, a layer of quicklime was placed at the bottom of the pit, the carcasses were then laid down, covered with another layer of quicklime, and the pit was closed with soil (Fig. 2). The protocol provided that the total quantity of lime had to be equal to 10 % of the total carcass weight. For sites 2, 3 and 4, a fence was installed around the pit. For site 1, the farmer built a dome of soil above the pit. More details about the sites and the buried animals are available in Table 1.

**Table 1:** Description of the burial sites.

Site ID	Species	Type of production	Number of carcasses and total weight	Time between clinical signs and burial
1	Broilers	Organic	8 000 broiler carcasses (2 buildings) 1 pit = 20 tonnes of carcasses	8 days
2	Pheasants and partridges	Breeders	14 000 pheasant carcasses and 18 000 partridge carcasses 1 pit = 30 tonnes of carcasses	6 days
3	Pheasants and partridges	Breeders	21 000 pheasant and partridge carcasses 1 pit = 20 tonnes of carcasses	7 days
4	Broilers	Future breeders	8 900 broiler carcasses 1 pit = 20 tonnes of carcasses	8 days
5	Broilers	Standard	48 000 broiler carcasses (3 buildings) 3 pits = 80 tonnes of carcasses	1 day

### Follow-up protocol and sample collection

The burial sites included in the study were selected by the veterinary services. The sites were chosen on the basis of logistical criteria (stakeholders’ responsiveness, farm accessibility, distance) and, above all, on the basis of the acceptance of the farmer, who was not necessarily ready to participate in a scientific study in the context of a major crisis. Each site was visited at least four times over a one-year period: one day before burial, the day of burial, and then 15 days, 2 months, 6 months, 9 months and/or 12 months after burial. At each visit, the protocol included taking soil samples (25 g each) and swabs, half of them on the pit and the others one meter from the of edge of the pit. The soil was sampled at a depth of around 15 cm with three samplings being then pooled in one pot of 120 mL and the swabs were made by going back and forth on each side of the pit. Only one of the farms (farm 5) used drilling water. All the others were connected to the public water supply. For this reason, water samples were only taken on this farm. At each visit on this farm, 500 mL borehole water samples were taken to assess possible water contamination. During the first visit, swabs on carcass feathers and leachate sample (120 mL) were done on site 1. Finally, the day of burial (visit 2) air samples were taken using a cyclone-based bioaerosol sampler, Coriolisµ microbial air sampler (Bertin Technologies, Montigny-le-Bretonneux, France): 300 L/min, 10 min/sample, in 10 to 12 mL of 0.005 % Triton X-100 solution (Sigma-Aldrich, Saint-Quentin-Fallavier, France) prepared in demineralized water and placed into a sterile sampling cone in order to measure viral dispersion. Moreover, visual observations of the pit and the surroundings were made and photographs were taken. The detailed follow-up protocol is presented in Table 2.

**Table 2:** Observations and samples protocol of the five burial sites.

Site ID	1 day before burial	Day of burial	15 days AB	2 months AB	6 months AB	9 months AB	12 months AB
1	Soil [2] Swabs on/near the pit [1] Swabs on carcasses pit [1] Leachate [1]	Swabs on/near the pit [1] Swabs on the pathway [1] Air [2]	Soil [8] Swabs on/near the pit [6]	Soil [2] Swabs on/near the pit [4]	Soil [2] Swabs on/near the pit [4]	Soil [2] Swabs on/near the pit [4]	Soil [2] Swabs on/near the pit [4]
2			Soil [8] Swabs on/near the pit [6]	Soil [2] Swabs on/near the pit [4]	Soil [2] Swabs on/near the pit [4]	Soil [2] Swabs on/near the pit [4]	Soil [2] Swabs on/near the pit [4]
3			Soil [8] Swabs on/near the pit [6]	Soil [2] Swabs on/near the pit [4]	Soil [2] Swabs on/near the pit [4]	Soil [2] Swabs on/near the pit [4]	Soil [2] Swabs on/near the pit [4]
4			Soil [8] Swabs on/near the pit [6]	Soil [2] Swabs on/near the pit [4]	Soil [2] Swabs on/near the pit [4]	Soil [2] Swabs on/near the pit [4]	Soil [2] Swabs on/near the pit [4]
5			Soil [8] Swabs on/near the pit [6] Drilling water [4]	Soil [2] Swabs on/near the pit [4] Drilling water [2]	Soil [2] Swabs on/near the pit [4] Drilling water [2]		Soil [2] Swabs on/near the pit [4]

AB: after burial.

### Microbiological, molecular, physical and chemical analyses

All samples were stored frozen before analysis. Microbiological analyses were performed on the collected samples to detect AIV genome, *Clostridium botulinum* and *Salmonella spp.* In addition, sampled drilling water was analysed to describe its physical and chemical properties (pH, conductivity at 25°C, ammonium, nitrates, nitrites, orthophosphate, chemical oxygen demand, biochemical oxygen demand, Kjeldahl nitrogen, total nitrogen, phosphorus and suspended matter).

### AIV genome detection

Boot swabs were suspended in 70 mL of phosphate-buffered saline buffer (PBS) (Sigma, St. Louis, MO, United States) and manual homogenisation was performed for at least 1 min. After centrifugation (1 500 x *g*, 4°C, 10 min), 100 µL of the supernatant were used for RNA extraction. The leachate was directly centrifuged (1 500 x *g*, 4°C, 10 min) and 100 µL of the supernatant were used for RNA extraction. For soil samples, 500 mg were collected and homogenised in 2 mL of PBS (Sigma) before being centrifuged (1 500 x *g*, 4°C, 10 min). For water samples, 100 µL were directly collected for RNA extraction.

All RNA extractions were performed with a NucleoMag VET kit (Macherey-Nagel, Düren, Germany) from 100 µL of sample using the KingFisher™ Flex System (Thermo Scientific, Waltham, MA, United States). In each sample, 10 µL of an internal positive control (IPC) were added to evaluate the presence of PCR inhibitors (Cherbonnel et al., 2013). Briefly, 300 µL of lysis buffer (RLT) (QIAGEN, Courtaboeuf, France), 3 µL of ß-mercaptoethanol (Sigma) and 4 µL of carrier were added to each sample and then agitated for 15 min at room temperature. After this step, 20 µL of magnetic beads (Macherey-Nagel) and 400 µL of isopropanol (Sigma) were added to each sample before being agitated for 5 min at room temperature. After addition of 600 µL of VEW1 buffer (Macherey-Nagel), 600 µL of VEW2 buffer (Macherey-Nagel) and 600 µL of 80 % ethanol (Sigma), the RNA was eluted in 100 µL of VEL buffer (Macherey-Nagel). To detected AIV genome, a real-time RT PCR targeting the M gene (M-rRT-PCR) was carried out using a commercial kit (TaqMan one-step rRT-PCR master mix; Applied Biosystems, Waltham, MA, United States) and the Applied Biosystems (ABI) 7500 Fast real-time PCR system. Concentrations of primers and probe and cycle conditions were used as described by (Spackman et al., 2002).

### Clostridium botulinum detection

For *C. botulinum*, samples were incubated at 70°C for 1 h before weighing, so as to inactivate any residual AIV. Only spores are detected using this protocol. Swabs were diluted in 250 mL pre-reduced trypticase-peptone-glucose-yeast extract (TPGY) (Skarin et al., 2010). Soil samples (25 g) were ten-fold diluted in pre-reduced TPGY. Leachate and drilling water were two-fold diluted in double concentrated TPGY. All were homogenised for 15 s using a Pulsifier® (Microgen, Camberley, United Kingdom) and incubated for at least 18 h at 37°C under anaerobic conditions. After incubation, 1 mL of each enrichment broth was collected. Cells were pelleted by centrifugation and subjected to DNA extraction using a DNeasy Powersoil Pro kit (QIAGEN, Courtaboeuf, France), according to the manufacturer's instructions. Genes encoding BoNT- A, B, E and F as well as the non-toxic non-haemagglutinin (*ntnh*) gene present within the neurotoxin gene cluster of *C. botulinum* group III, i.e. *C. botulinum* C, D, C/D and D/C, were detected using PCR. Real-time PCR using a Bio-Rad CFX96 thermal cycler (Bio-Rad, Marne-la-Coquette, France) was used to detect the target in samples, with 5 μL DNA template, 10 μL PerfeCTa® qPCR ToughMix (VWR International SAS, Briare, France) and primers and probes (Fach et al., 2009; Woudstra et al., 2015) as previously described in Le Maréchal et al. (2024)

### Salmonella detection

The detection of *Salmonella* spp. by culture was carried out in accordance with the French Standard NF U 47-100 (Methods of analysis in animal health - Research by isolation and identification of any serovar or specified serovar(s) of *Salmonella* in the animal production environment (NF U 47-100 - juillet 2007, 2007,)).

## Results

### Visual observations

Results of the visual observations are presented in Table 3. For all sites but one (site 1), cracks appeared quickly after burial, but with no major further changes. In the case of site 1, cracks did not appeared due to the dome of soil but the pit raised. The anomalies noted in terms of changes to the pits over time were subsidence, the presence of traces of wild and domestic animals (droppings, holes and burrows), and the rising to the surface of pieces of carcasses and feathers (Table 3, N file 1). Vegetation gradually developed on all burial sites, making them less and less visible.

**Table 3:** Summary results of visual observations on the five burial sites over the first three months.

Site ID	After 15 days	After 2 months
1	No fencing Slight presence of lime Raised pit (mound)	Regrowth of vegetation Passage of wild animals visible Pieces of carcass
2	Closed site Presence of lime Flat surface	Passage of wild animals visible (numerous burrows, droppings) Cracks several meters long (5 – 6 m)
3	Closed site Presence of lime Flat surface	Regrowth of vegetation Cracks
4	Closed site Presence of lime Flat surface Cattle nearby Passage of wild animals visible (presence of molehills and wild animal tracks)	Cracks Manure pile nearby
5	No fencing Presence of lime Collapse of the pit (rising carcasses a few days after burial)	Regrowth of vegetation Presence of flies Pieces of carcass (bones and feathers)

### Microbiological results

#### Avian influenza virus

Results for the detection of AI viral genome (M gene) are presented in Table 4 and Supplementary file 2. Of the 201 samples analysed, 8 (4 %) were found to have AIV genome. Virus genome was found at all burial sites. Positive results were found up to 9 months after burial on one farm (Site 3). However, the results show a low level of viral RNA, given the late Ct values obtained (37–39) after burial, (see Supplementary file 2). AIV was mainly detected in swabs samples (4/8), and to a lesser extent in soil (3/8) and leachate (1/8). All air samples tested negative.

**Table 4:** Summary results of microbiological analyses conducted on samples taken at the five burial sites over a one-year period.

Site ID	1 day before burial	Day of burial	15 days AB	2 months AB	6 months AB	9 months AB	12 months AB
1	AIV [+] *Salmonella* spp. [-]	AIV [-] *Salmonella* spp. [-]	AIV [-] *C. botulinum* B [+] *Salmonella* spp. [-]	AIV [-] *C. botulinum* B [+] *Salmonella* spp. [-]	AIV [-] *C. botulinum* B [+] *Salmonella* spp. [-]	AIV [-] *C. botulinum* B [+] *Salmonella* spp. [-]	AIV [-] *C. botulinum* A [+] *Salmonella* spp. [-]
2			AIV [-] *C. botulinum* B, GIII [+] *Salmonella* spp. [-]	AIV [-] *C. botulinum* B [+] *Salmonella* spp. [-]	AIV [+] *C. botulinum* B [+] *Salmonella* spp. [-]	AIV [+] *C. botulinum* [-] *Salmonella* spp. [-]	AIV [-] *C. botulinum* B [+] *Salmonella* spp. [-]
3			AIV [+] *C. botulinum* B [+] *Salmonella* spp. [-]	AIV [+] *C. botulinum* A, B [+] *Salmonella* spp. [-]	AIV [-] *C. botulinum* B [+] *Salmonella* spp. [-]	AIV [-] *C. botulinum* A*,* B [+] *Salmonella* spp. [-]	AIV [-] *C. botulinum* B [+] *Salmonella* spp. [-]
4			AIV [-] *C. botulinum* B [+] *Salmonella* spp. [-]	AIV [+] *C. botulinum* [-] *Salmonella* spp. [-]	AIV [-] *C. botulinum* [-] *Salmonella* spp. [-]	AIV [-] *C. botulinum* [-] *Salmonella* spp. [-]	AIV [-] *C. botulinum* [-] *Salmonella* spp. [-]
5			AIV [+] *C. botulinum* [-] *Salmonella* spp. [-]	AIV [-] *C. botulinum* [-] *Salmonella* spp. [-]	AIV [-] *C. botulinum* [-] *Salmonella* spp. [-]		AIV [-] *C. botulinum* [-] *Salmonella* spp. [-]

AB: after burial.

[+] Positive results.

[-] Negative results.

### Clostridium botulinum

Of the 117 samples analysed, 19 (16 %, Table 4) showed the presence of *C. botulinum. Clostridium botulinum* A, B as well as *C. botulinum* group III (i.e. C, D, C/D or D/C) were detected at four sites out of five and in various samples up to one year after burial (3 out of 5). Contrary to what was obtained for AIV testing, *C. botulinum* was detected preferentially in soil samples (18/19) rather than in swabs (1/19) (Supplementary file 2).

### Salmonella spp

All samples tested negative (0/109) for *Salmonella* spp. at all sites and at all sampling points (Table 4).

### Physical and chemical characteristics of drilling water

All drilling water samples taken from Site 5 were compliant with drinking water standards. Detailed results are presented in Supplementary file 3.

## Discussion

The European Union is one of the world's largest poultry producers with more than 13.4 million tons produced annually. France is the third contributor with 11 % of the European poultry production (https://agriculture.ec.europa.eu). In the recent years, both the European and the global poultry sector faced several major HPAI outbreaks. Like many other countries, France was hard hit by these epizootics. In this context, finding new options to handle carcasses of infected animals as quickly as possible was necessary.

The main contribution of our study lies in the follow-up of five on-farm burial sites in real-world conditions of HPAI outbreaks. The duration of the study made it possible to obtain valuable data on the visual changes to the pits and the microbiological and chemical characteristics of their immediate environment on a long-term basis. The large number of samples taken from different environments (air, water, soil and swabs) provided us with an exhaustive view of the surrounding ecosystem. In the literature, it is often the chemical properties of borehole water around the pits that are assessed (Glanville, 2000; Hseu and Chen, 2017; Kaown et al., 2015; Koh et al., 2019; Kwon et al., 2017; Ritter and Chirnside, 1995). In this project, we also focused on the microbiological quality of the pit's environment. Of note, while many studies have assessed the presence of faecal contamination indicators (e.g. coliforms), we focused on pathogens of high interest in the context of poultry production and HPAI outbreaks: AIV itself, *Clostridium botulinum*, a spore-forming bacterium for which animal carcasses are optimal for its growth and botulinum neurotoxin production (Meurens et al., 2023), and *Salmonella* spp. which are both an indicator of faecal contamination and bacteria of interest in terms of animal and human health. Moreover, the study was conducted in close collaboration with local veterinary authorities, farmers and hydrogeologists, which enabled us to choose appropriate burial sites to design a feasible protocol and to collect data in the field ourselves. However, this paper is not derived from a research project per se, but from operational fieldwork in a crisis situation. As such, the study has some limitations. Firstly, only five burial sites were followed-up, which prevented us from performing statistical analysis. Moreover, only one site was followed up from the very beginning of the burial process, whereas the four others were followed up from day 15 only. On one farm (Site 5), it was not possible to collect samples at nine months after burial due to an HPAI outbreak nearby. Secondly, air and drilling water samples were not systematically collected at all sites, but we were able to carry out complete follow-up with drilling water samples at one site.

The results of this study will first of all enable us to reflect on burial site construction processes and the critical points to be respected. Burial sites were constructed within a short period of time to prevent the rapid spread of HPAI outbreaks. It was very important to proceed as quickly as possible to carcass disposal. Delays between detection of infection and culling and carcass disposal may raise concerns in terms of viral spread, especially with an infectious agent that is capable of airborne propagation (Gwyther et al., 2011). Ideally, burial sites should be identified in ‘peace-time’, i.e. before any outbreak (Dolan and Koppel, 2005). This was not the case in the situation described. We recommend that burial operations be planned in advance as much as possible and to integrate a burial protocol in the farm's biosecurity plan. This requires a paradigm shift from a one-off crisis management approach to an organisation that is permanently capable of dealing with repeated, high-frequency crises or crises of a new nature. In this study, hydrogeologists were asked to examine the farms’ locations and characteristics, and to give their opinion about the farms’ suitability for burial. As recommended in several guidelines, the distance to drinking water supply, human habitations, coast, surface water, as well as soil characteristics were taken into account in this decision (Australia, 2021; Consortium, 2004). In terms of type and size of pits, the burial sites included in this project were rectangular trenches, which is in line with current recommendations (Chowdhury et al., 2019). As regards the depth of trenches and pits, different recommendations were found. Antea (2010) recommend not digging deep trenches so that the carcasses are in the biologically active part of the soil. Importantly, carcass decomposition is slowed down when burial is deep (over one metre) due to the absence of scavengers and insects, low soil temperatures and the absence of oxygen (Dent et al., 2004; Janaway, 1997; Pawlett et al., 2024; Polson et al., 1985; Pawlett et al., 2018). In our study, visual monitoring showed how the sites changed over the course of a year. The most frequent observations that persisted over time were cracks and subsidence at certain sites, and even the “rising” of carcasses at one of them. It is likely that the cracks appeared as a result of the pile settling due to the loss of volume of the carcass over time but lack of venting the pits was also considered. A more detailed analysis of the type of soil may be requested to ensure pit stability, biosecurity and leachate containment in the long term (Chowdhury et al., 2019). Moreover, to avoid cracks and carcass rising as observed in our study, we recommend not digging deep trenches (< 1.3 m) and to add mounding soil to the top of the pit, as carried out at Site 1 and described in Chowdhury et al. (2019). The “rising” of carcasses is probably due to the production of gas during the decomposition of buried carcasses trapped inside the pit. In order to manage gas produced, mass burial facilities usually include gas collection system (Chowdhury et al., 2019).

The presence of traces of wild and/or domestic animals, certainly attracted by the buried poultry carcasses, was also noted throughout the period (burrows, holes, droppings and footprints). Remains of carcasses (decomposing animals, bones, feathers, etc.) were identified at most of the sites. This leads us to recommend better fencing of the sites to prevent access by wildlife and livestock. The last point related to the design of burial pits relates to the use of lime. Few scientific articles have reported the effects of lime on whole animal carcasses. Nevertheless, a number of studies conducted under different conditions have shown that the addition of lime effectively reduces pathogen survival and thus the risk of pathogen spread in the environment and to other farms (Avery et al., 2009; Lopes et al., 2013; Sánchez et al., 2008; Tanaka et al., 2014). However, adding lime to carcasses leads to a delay in carcass degradation, in relation to the impact of lime on the microbial communities associated with carcass decomposition (Schotsmans et al., 2014, et al., 2014). As a result, existing recommendations in the literature vary and are sometimes contradictory. In these conditions, the situations in which the use of lime would be necessary are currently not clearly known. Studies and research aimed at identifying these situations, specifying the conditions of use of lime, and assessing its environmental impact are needed to provide concrete answers to this point.

In addition to these points related to the burial protocol, there were limitations in the follow-up protocol. Firstly, it was not possible to install temperature sensors in the pit due to the emergency context. In the future, it would be interesting to include pit temperature monitoring measurements in the burial protocol to assess the sanitising effect of the process. Similarly, it was not possible to take samples over time by digging inside the pit. At all sites but one, no samples were collected on or before the day of burial. This was due to the urgent nature of the burial, which was carried out in a crisis situation with no pre-established protocol. Secondly, microbiological analyses to detect *Salmonella* spp. and *Clostridium botulinum* could only be carried out once the samples had tested negative for avian influenza, requiring a freezing step before analysis. Although this freezing step is recommended for the detection of *Clostridium botulinum*, it may reduce the detection of *Salmonella* spp. in the samples. Thus, *Salmonella* testing should be interpreted carefully because stressed *Salmonella* and/or *Salmonella* in low numbers in a sample need a longer latency period than that of the associated flora to begin their growth.

Again, this shows the importance of preparing a robust protocol with a sufficient number of samples and good reactivity in analysing the samples. In terms of microbiological changes over time, it is interesting to note that AIV was found in only 4 % of the collected samples, but at all burial sites. Our results showed that AIV RNA can persist for around 9 months in the environment of the burial pit, which justifies restricting access to the site by people and animals for several months. However, we did not evaluate the infectivity of the viral genome found in the samples, and these five farms did not experience HPAI resurgence in the following crisis. Additionally, *C. botulinum* was detected in more samples (16 %), at four sites out of five, and up to one year after burial. *Clostridium botulinum* type B was detected in particular (14 %). This type is commonly detected in soils (Palmer et al., 2019), notably in France (Le Maréchal et al. submitted). No studies have been conducted up to now to evaluate the carriage of *C. botulinum* type B by poultry. Carriage has, however, been reported for pigs and cattle (Dahlenborg et al., 2001, 2003; Myllykoski et al., 2006), raising the question of the possibility of carriage by other animal species and its origin here. The absence of sampling at day 0 made it impossible to determine the initial contamination of the sites prior to burial. Based on available data, the most likely hypothesis is that the spores were already present in the soil, although it is not possible to rule out a source linked to poultry carcasses themselves. Nonetheless, the presence of the bacterium in conditions favourable for its development could therefore be the cause of secondary contamination of the environment. This information underlines the importance of fencing off burial sites and moving them away from areas where livestock are reared and grazed, particularly poultry and cattle, as well as areas where crops are grown for human and animal consumption, in order to avoid secondary contamination and possible cross-contamination. With regard to water analyses, monitoring at Site 5 showed that the water complied chemically with drinking water standards. In contrast to certain previously published studies (Glanville, 2000; Hseu and Chen, 2017; Kaown et al., 2015; Kim, and Kim, 2012; Koh et al., 2019; Kwon et al., 2017; Ritter and Chirnside, 1995), we did not observe any increase in parameters such as nitrates, nitrites or phosphorus over time. It would be interesting to carry out other analyses on the samples taken, for example to look for chemical contaminants such as veterinary drug residues, and to look for resistance plasmids.

In conclusion, at first sight, this method of on-farm burial appears to be simple, quick and cheap. However, our study highlights the need for pre-established and strict protocols, for both burial itself and monitoring, in order to limit environmental risks and pathogen persistence. As such, our crisis experience will prove invaluable in using operational epidemiology to better prepare for future emergency settings, and this study can be used as a baseline for preparing better burial and follow-up protocols. Assessments revealed how these burial pits changed over time and the need to monitor them regularly so that corrective measures can be taken if necessary (restabilising the pits, fencing the sites, keeping livestock away, etc.). Therefore, on-farm burial should be included in the farm's biosecurity plan as well as in the national contingency plan. Farmer perceptions, health and safety should also be taken into account in the burial decision and the protocol itself. Alternatives are described in the literature as being safer for public health risks (Pollard et al., 2008). Among them, carcass composting appears to be a promising option that is already used in several countries (United States, Canada and Australia), but it is not authorised in the European Union. More research is needed in this area to investigate the sanitising effect of composting as well as the technical, social and economic feasibility of this method.

## Declaration of competing interest

The authors declare that they have no known competing financial interests or personal relationships that could have appeared to influence the work reported in this paper.

## Appendix Supplementary materials

Image, application 1

Image, application 2
