# Supplementary material for: Burying poultry carcasses on farms as a disposal option in crisis situations: learnings and perspectives from a field study during an avian influenza epizootic in France
Source: Poult Sci. 2025 Jan 13;104(2):104806. doi: 10.1016/j.psj.2025.104806 (PMC11786764; doi:10.1016/j.psj.2025.104806)
Supplement: Supplementary file 1 [file mmc1.docx]

**Supplementary files**

**Supplementary file 1. Photographs of the burial sites**

1. **Example of a burial pit: in process (site 1)**

**
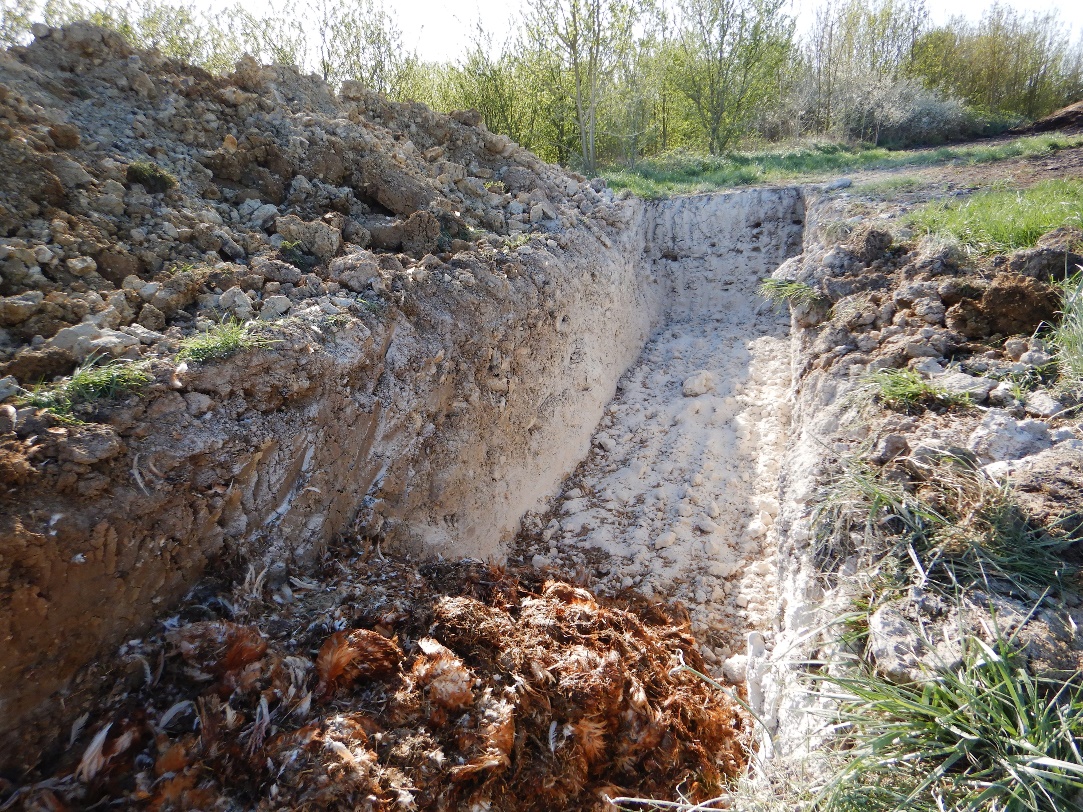
**

1. **Example of a burial pit with a fence: end of the process (site 2)**

**
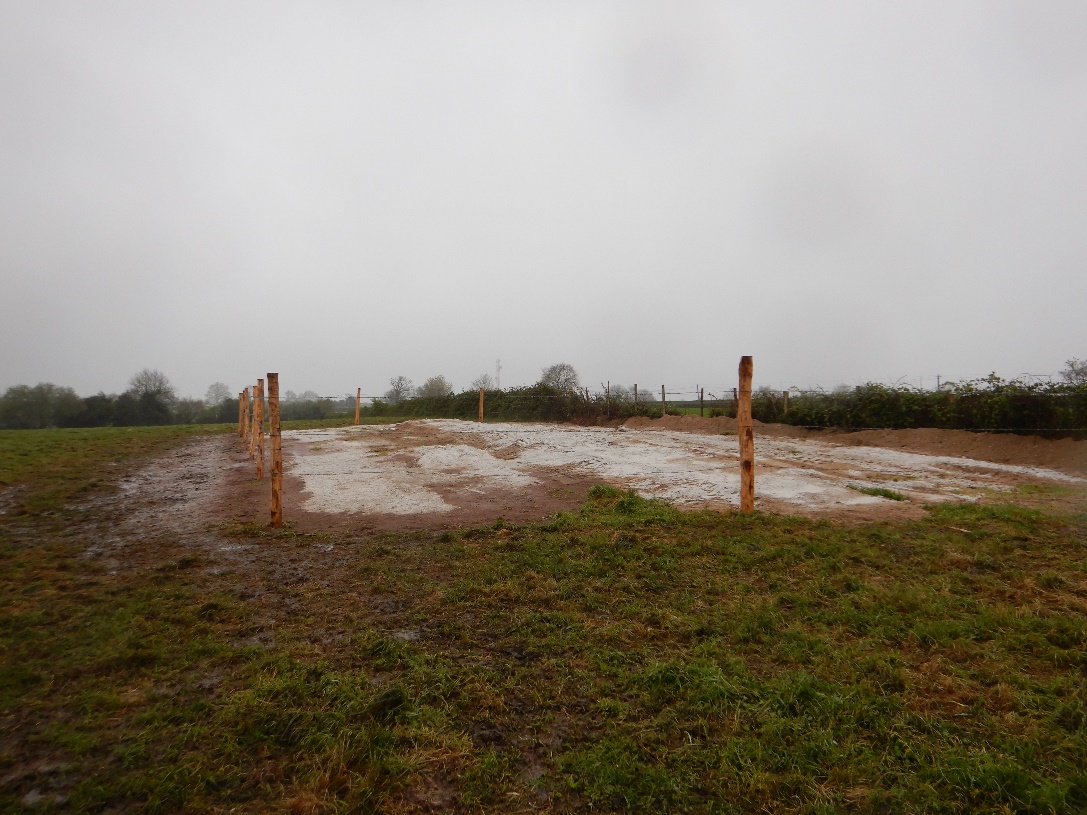
**

1. **Changes observed in a burial pit: example of a crack (6 x 0.12 m) (site 4)**

**
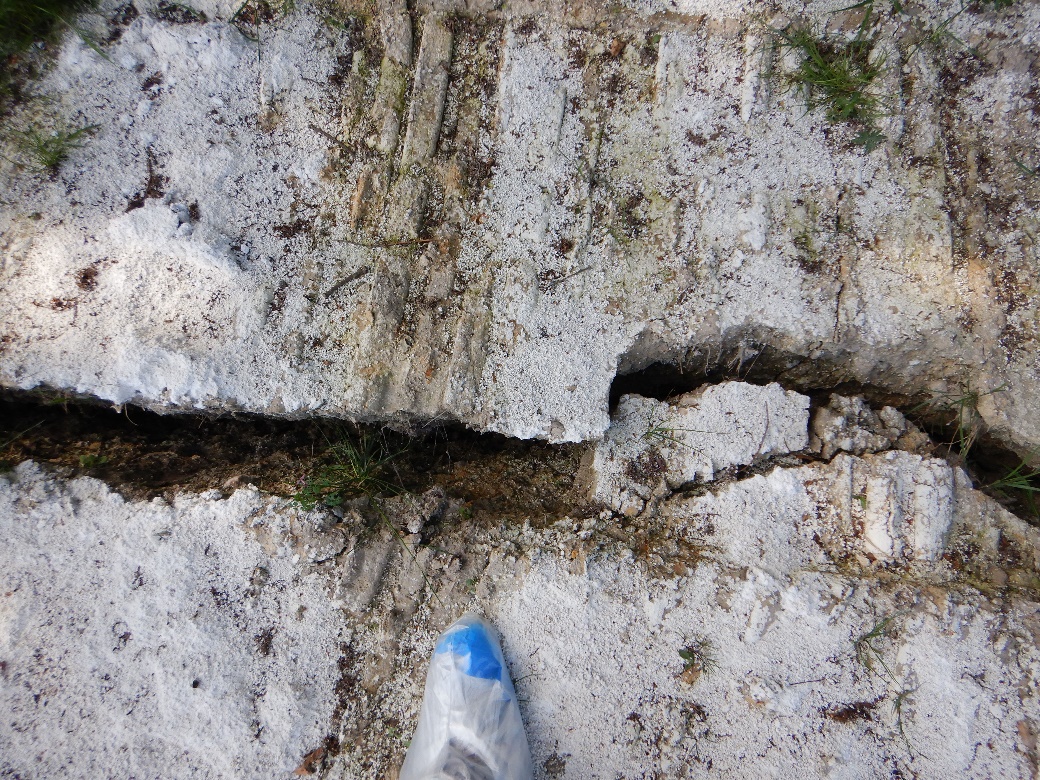
**

1. **Changes observed in a burial pit: example of carcass pieces rising to the surface after 2 months (site 5)**

**
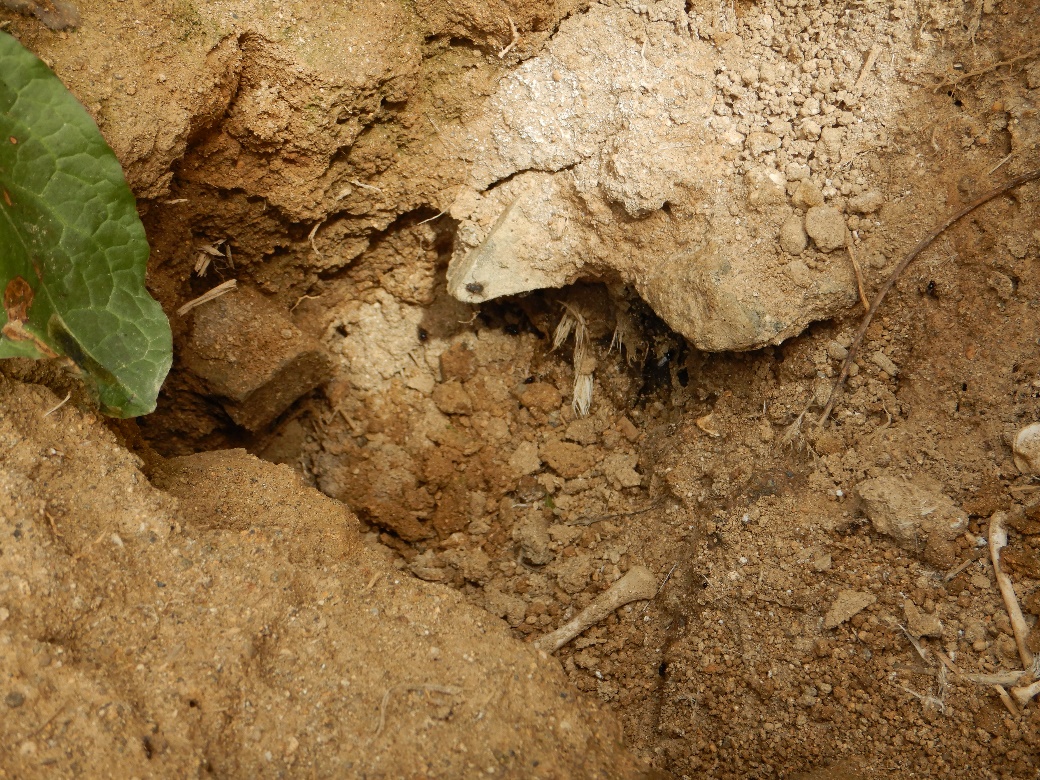
**

Piece of carcass with feathers

Poultry bones

Flies

1. **Changes observed in a burial pit: vegetation covering the pit after 6 month (site 3)**

**
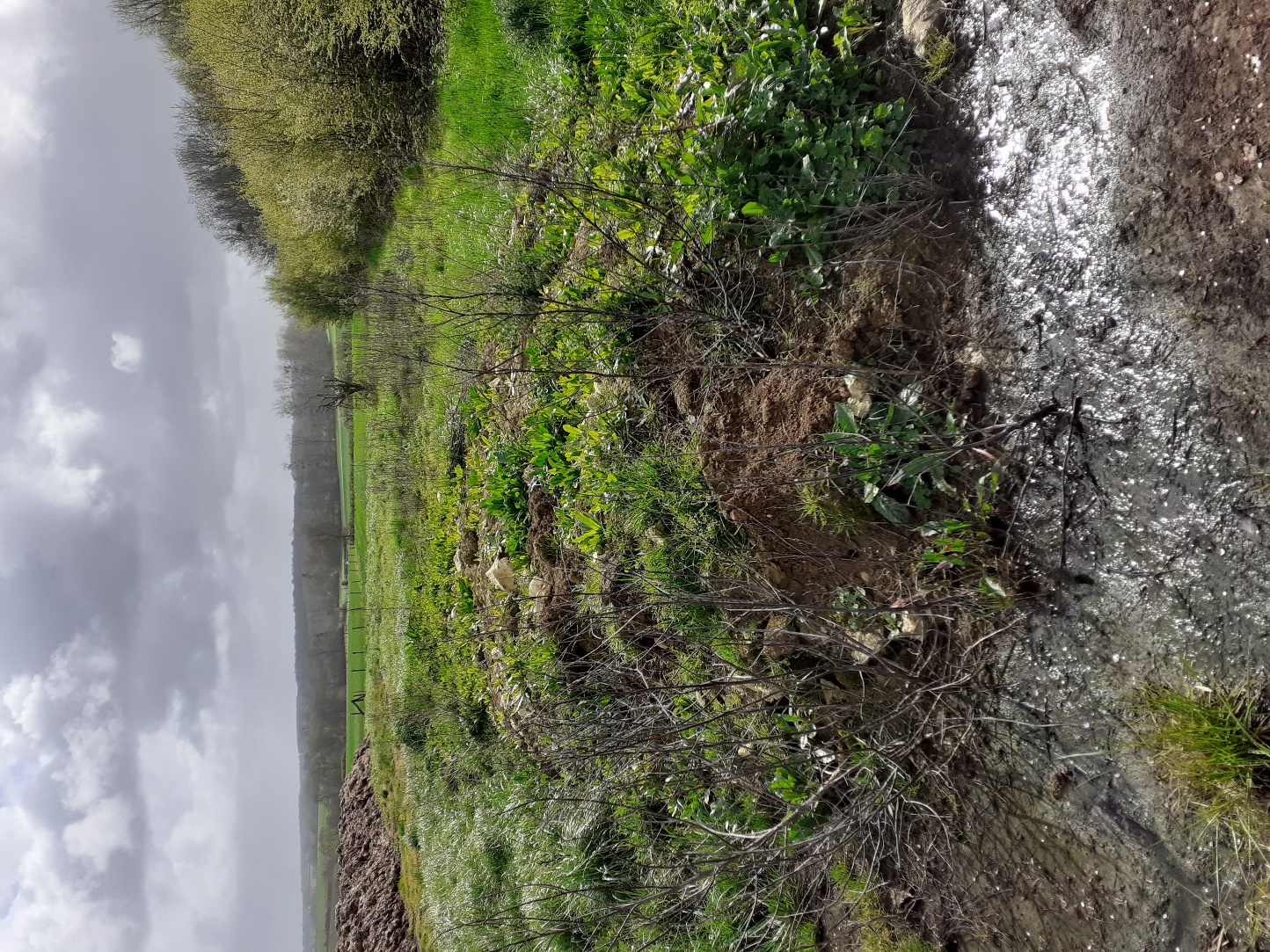
**

**Supplementary file 2. Microbiological results related to the detection of avian influenza virus and *Clostridium botulinum* in various samples collected at five burial sites of poultry carcasses**

Separate Excel file

**Supplementary file 3. Physical and chemical analysis of drilling water samples (site 5)**

|  | 15 days after burial | 2 months after burial | 6 months after burial |
| --- | --- | --- | --- |
| pH | 6.4 | 6.3 | 6.1 |
| conductivity at 25°C (μS/cm) | 281 | 284 | 280 |
| ammonium (mg(NH4)/L) | < 0.010 | < 0.010 | 0.012 |
| nitrates (mg/L) | 14 | 14 | 15 |
| nitrites (mg(N)/L) | < 0.003 | < 0.003 | < 0.003 |
| orthophosphate (mg(PO4)/L) | 0.084 | 0.098 | 0.093 |
| chemical oxygen demand (mg/L O_2_) | < 10 | < 10 | < 10 |
| biochemical oxygen demand (mg(O_2_)/L) | 0.61 | < 0.50 | < 0.50 |
| Kjeldahl nitrogen (mg(N)/L) | < 0.42 | < 0.42 | < 0.42 |
| total nitrogen (mg/L) | 14.00 | 14.00 | 15.00 |
| phosphorus (mg/L) | 0.025 | 0.038 | 0.031 |
| suspended matter (mg/L) | < 2 | < 2 | < 2 |
